# Mutation of Kinesin-6 *Kif20b* causes defects in cortical neuron polarization and morphogenesis

**DOI:** 10.1186/s13064-017-0082-5

**Published:** 2017-03-31

**Authors:** Katrina C. McNeely, Timothy D. Cupp, Jessica Neville Little, Kerstin M. Janisch, Ayushma Shrestha, Noelle D. Dwyer

**Affiliations:** Department of Cell Biology, University of Virginia, Charlottesville, VA 22908 USA

**Keywords:** Kinesin, Kif20b, Axon outgrowth, Microtubule, Mouse, Apical dendrite, Axon branching, Cytoskeleton, Filopodia, Microcephaly

## Abstract

**Background:**

How neurons change their cytoskeleton to adopt their complex polarized morphology is still not understood. Growing evidence suggests that proteins that help build microtubule structures during cell division are also involved in building and remodeling the complex cytoskeletons of neurons. Kif20b (previously called MPP1 or Mphosph1) is the most divergent member of the Kinesin-6 family of “mitotic” kinesins that also includes Kif23/MKLP1 and Kif20a/MKLP2. We previously isolated a loss-of-function mouse mutant of *Kif20b* and showed that it had a thalamocortical axon guidance defect and microcephaly.

**Methods:**

We demonstrate here, using the mouse mutant, that *Kif20b* is required for neuron morphogenesis in the embryonic neocortex. In vivo and in vitro cortical neurons were labeled and imaged to analyze various aspects of morphogenesis.

**Results:**

Loss of *Kif20b* disrupts polarization as well as neurite outgrowth, branching and caliber. In vivo, mutant cortical neurons show defects in orientation, and have shorter thinner apical dendrites that branch closer to the cell body. In vitro, without external polarity cues, *Kif20b* mutant neurons show a strong polarization defect. This may be due in part to loss of the polarity protein Shootin1 from the axonal growth cone. Those mutant neurons that do succeed in polarizing have shorter axons with more branches, and longer minor neurites. These changes in shape are not due to alterations in cell fate or neuron layer type. Surprisingly, both axons and minor neurites of mutant neurons have increased widths and longer growth cone filopodia, which correlate with abnormal microtubule organization. Live analysis of axon extension shows that *Kif20b* mutant axons display more variable growth with increased retraction.

**Conclusions:**

These results demonstrate that *Kif20b* is required cell-autonomously for proper morphogenesis of cortical pyramidal neurons. *Kif20b* regulates neuron polarization, and axon and dendrite branching, outgrowth, and caliber. Kif20b protein may act by bundling microtubules into tight arrays and by localizing effectors such as Shootin1. Thus it may help shape neurites, sustain consistent axon growth, and inhibit branching. This work advances our understanding of how neurons regulate their cytoskeleton to build their elaborate shapes. Finally, it suggests that neuronal connectivity defects may be present in some types of microcephaly.

**Electronic supplementary material:**

The online version of this article (doi:10.1186/s13064-017-0082-5) contains supplementary material, which is available to authorized users.

## Background

The function of the nervous system depends on neurons having a receiving end (dendrites) and a transmitting end (a single axon). A cortical pyramidal neuron grows a large apical dendrite at the apex of the cell body, several shorter basal dendrites, and a single axon from the base to connect to distant targets. Early polarization occurs soon after the neuron’s birth, during the migratory phase through the intermediate zone. The newborn neuron first becomes multipolar with several short neurites, and then becomes bipolar with a leading process oriented outward in the direction of migration away from the ventricle. As it migrates, it extends an axon from the trailing end, but does not initiate dendrite growth until it finishes migrating.

Early polarization events can be modeled in dissociated cultures of embryonic hippocampal or cortical neurons [1]. During repolarization in vitro, the neurons first appear round and flat like a fibroblast (Stage 1, unpolarized). Within a day they begin to extend several undifferentiated neurites of about equal length (Stage 2, Multipolar). The growing neurites are full of microtubules, and they are tipped by actin-rich growth cones with lamellae and filopodia. In a stochastic manner, one of the neurites elongates rapidly to become the axon (Stage 3, Polarized). The remaining neurites become dendrites and remain shorter and thicker than the axon. This invaluable system has revealed many factors important for polarization [2], but the cytoskeletal mechanisms underlying the development of complex neuronal morphology are still not well understood [3, 4].

Various kinesin motor proteins are crucial for establishing or maintaining neuronal polarity and structure through their interactions with microtubules and cargoes [5]. The Kinesin-6 subfamily members are plus-end- directed microtubule motors known as “mitotic” kinesins for their roles in cytokinesis but some may have additional functions [6]. Mammalian cells express three Kinesin-6 family members: Kif23/MKLP1, Kif20a/MKLP2, and Kif20b (formerly called mitotic phospho-protein 1, MPP1 or Mphosph1). RNAi of *Kif23* in cultured rat sympathetic neurons disrupted microtubule polarity of dendrites and resulted in longer axons and dendrites [7, 8]. Mutation of the *Drosophila Kif23* ortholog, *pavarotti (pav)*, caused excessive microtubule sliding, axon growth, and axon branching [9, 10].

The third member of the mammalian Kinesin-6 subfamily, Kif20b, is less understood and has higher molecular weight and lower abundance than Kif23 or Kif20a. Interestingly, it has a two-fold longer stalk comprised of four coiled-coil domains linked by three hinges [11, 12]. In in vitro assays, KIF20B was sufficient to both slide and bundle microtubules in an ATP-dependent manner [11]. In a previous mouse ENU screen, we found a recessive, neonatal lethal mutant that displayed microcephaly and axon guidance defects in a subset of thalamocortical axons at embryonic day (E) 18.5 [13]. Genetic mapping and a complementation test identified *Kif20b* as the mutant gene [14]. The point mutation in *Kif20b* causes an mRNA splicing error, a consequent frameshift and premature termination codons, and reduces Kif20b protein to undetectable levels on immunoblots or cell staining [14]. Mutants show cytokinesis defects in neural stem cells of embryonic cortex. Cortical size and thickness is reduced in the *Kif20b* mutants due to decreased numbers of neurons and intermediate progenitors. Despite this, laminar organization and most axon tracts appear grossly normal at birth [13, 14].

Here, to explore *Kif20b* roles in neuronal development during corticogenesis, we took advantage of this genetic loss-of-function mutant. In *Kif20b* mutant brains, cortical pyramidal neurons have shorter, thinner, apical dendrites which branch closer to the cell body, extra axon branches, and are sometimes misoriented. To separate non- cell-autonomous effects, we pursued further analyses in dissociated neuron cultures. Surprisingly, when isolated from their normal brain environment, *Kif20b* mutant neurons show a strong polarization defect. This defect may be at least partly explained by a role for Kif20b in localizing the polarity protein Shootin1. The polarization defect is not due to cell fate changes and affected both deep and superficial layer types. Furthermore, the *Kif20b* mutant neurons that do successfully polarize have a variety of morphological changes including shorter, more branched axons and longer minor neurites. Mutant neurites are wider, and growth cone filopodia are longer with increased microtubule penetration. In live imaging, axons of *Kif20b* mutant neurons appear to pause less and retract more. These data indicate that Kif20b is important for polarization and maintaining axon growth and preventing branching, and suggest that it acts both by localizing cargo and organizing microtubule bundles.

## Methods

### Cell culture

To prepare for neuron culture and plating, 18 mm round coverslips were washed twice every 10 min with double-distilled, UV-irradiated water and treated in nitric acid overnight. Following three subsequent washes with double-distilled water, coverslips were placed in an oven at 160 °C overnight to dry and sterilize. After allowing to cool the next day, each coverslip was treated with 200 μL poly-L-lysine (PLL) solution (1 μg/mL in borate buffer) and incubated overnight at 37 °C. This was followed by double-distilled water washes (2 h washes were done twice following three quick rinses) and application of neuron plating media (.5 mL-1 mL). Neuron plating media is filter-sterilized and consists of 500 mL Minimum Essential Medium (MEM) with glutamine, 5 mL Penicillin/Streptomycin, 15 mL 20% glucose, 5 mL Sodium Pyruvate, and 10% Fetal Bovine Serum. At E14.5, pregnant females were sacrificed and the embryos placed into cold HBSS mix (500 mL Hank’s Balanced Salt Solution (HBSS) with 5 mL HEPES and 5 mL Penicillin/Streptomycin. Using fine forceps, pieces of cortex were collected and placed into a tube containing a 0.05% trypsin solution for 15 min in a 37 °C water bath. Following trypsin digestion, the resulting neuron pellets were rinsed 3 times every 5 min with HBSS mix and then treated with neuron plating medium during trituration. Appropriate volumes of the resulting solution of neuron plating medium and dissociated neurons were pipetted onto the PLL-coated coverslips to achieve a density of 50,000 cells/mL. Medium was switched from Neuron Plating Medium to Neurobasal and B27 (NB27) after three h. 48 h after being plated initially, the neurons were fixed in 2% Paraformaldehyde (PFA) for ten minutes and then in 2% PFA with 30% sucrose for 10 min. Finally, coverslips were rinsed in one time in Phosphate Buffer Saline (PBS) for 10 min three times and kept at 4 °C until ready for immunofluorescent staining.

### Immunocytochemistry

Following dissociation, neuron plating, and fixation, coverslips were incubated for an hour at room temperature in blocking buffer (0.1% Triton-X, 2% Normal Goat Serum in PBS) and then overnight at 4 °C or for 3 h at room temperature in appropriate primary antibody solution (antibody diluted in blocking solution). After primary incubation, coverslips were rinsed in PBS (3 times every 10 min) and then incubated at room temperature with appropriate secondary antibody solution (1:200 dilution) for 30 min in the dark. Following final washes in PBS, coverslips were mounted onto glass slides with Fluoromount.

### Antibodies

Primary antibodies used were a rabbit or mouse monoclonal antibody against neuron-specific beta-III tubulin (Tuj1) at a dilution of 1:500 in blocking buffer (Covance clone 1-15-79 D71G9 and MMS-435P, Abcam ab52623), a rat polyclonal antibody against Ctip2 at a dilution of 1:500 (Abcam ab18465), mouse tubulin-alpha clone (DM1alpha) at a dilution of 1:500 (Thermo Scientific MS-581-PO), a mouse monoclonal antibody against Tau at a dilution of 1:200 (Millipore mab2370), a rabbit polyclonal antibody against DCX at a dilution of 1:1200 (Abcam, ab18723) and (green)-Phalloidin (Molecular Probes O7466) at a dilution of 1:50. Species-specific secondary antibodies were conjugated to Alexa fluorophores (Invitrogen) at a dilution of 1:200 in blocking buffer. Shootin1 antibody (1: 100, B627, raised against the first 456aa) was a gift from Tamar Sapir and Orly Reiner, Weizmann Institute [15].

Multiple Kif20b antibodies have been verified for specificity by detecting signal in the midbodies of control dividing cells (HeLa and MEFs) and not in depleted or mutant cells. All of these antibodies were subsequently tried on mouse neurons. Published antibodies were kind gifts of Fabienne Pirollet (made to full length human KIF20B, [11]) and Orly Reiner (made to 1002 to 1442 of Kif20b, [15]). A commercially available monoclonal from Santa Cruz (raised against amino acids 1557–1675 of human KIF20B, catalog sc-515194) was tried. Both peptide anti-sera and purified antibodies to the N-terminal domain and C-terminal were also tried [14]. Multiple fixation conditions including TCA, PFA/MeOH, PFA only, permeabilized before fixing, and BRB80 then PFA were tried in combination with the previous mentioned antibodies. Due to large background to signal ratio no noticeable difference between control and *Kif20b* mutant neurons was detected with any of the antibodies or different fixation conditions.

### Image acquisition and analysis

Fluorescent images were obtained on a Carl Zeiss widefield epi-fluorescence microscope via AxioVision camera and software. Low-magnification images for stage analyses were taken at 20× while high-magnification images for neuron measurements were taken at 40× or 100×. Image analysis was completed through ImageJ software. We used the NeuronJ software plugin to trace and measure axons and minor neurites. Axon branches were only counted if they were a minimum of 5 μm. Neurite lengths were measured from the base of the process at the soma to the tip of the Tuj1 stain. Stage 3 neurons’ minor neurites were measured in thickness at 0 μm, 5 μm, and 10 μm from the edge of the soma. Stage 3 neurons’ axons were measured in thickness at 0 μm, 5 μm, 10 μm, and 25 μm from the edge of the soma. For Fig. 7D, images of tubulin in axons were captured using a Deltavision widefield microscope and then deconvolved. The intensity of tubulin staining was measured across the width of the axon at 5 μm, 10 μm, and 25 μm using Zeiss Zen 2 lite imaging software profile tool.

### Polarity stage analyses

Neurons were considered to be in Stage 1 if they extended broad lamellipodia with no clear, coalesced neurites. The neurites of Stage 2 neurons are all of similar length. A neuron was placed into the Stage 3 category when one neurite was at least twice as long as the next longest neurite. Neuronal protrusions were considered to be neurites or neurite branches if they had significant microtubule invasion.

### Measuring growth cones and filopodia

The growth cone area was measured from the base of intense phalloidin-stained actin at the axon neck, around the growth cone tracing lamellipodial edges. The lamellipodial edge was considered to be where the filopodium becomes uniform in width (the filopodial base). We considered filopodia to be actin protrusions extending out of lamellipodia at growth cones. Filopodial extensions generally have brighter phalloidin staining than neighboring lamellipodial edges. Measurements were taken from the filopodia tip to its base, where the edges begin to splay apart and become lamellae.

### Shootin1, DCX, and Tau localization measurements

Images of Shootin1 immunostaining were captured using the Zeiss AxioImagerZ1 Microscope with a 100× oil objective, and a constant 600 milliseconds exposure time. Tau and DCX images were captured using the 40× objective on the Zeiss Observer Z1 microscope with Axiocam 506 mono camera. Exposure time was kept constant (DCX 300 ms and Tau 200 ms). Zeiss Zen 2 lite imaging software profile tool was used to measure intensity. Excel was used to compare individual cells line scans as well as create averages across genotypes for comparison.

### RT-PCR

Mouse embryonic fibroblasts (from E14 mice) and mouse cortical neurons (4 DIV) were grown at a density of ~750000 cells/dish. RNA extraction of the harvested cells was done using the PureLink^TM^ RNA Mini Kit from life technologies according to the manufacturer’s handbook. RNA content in the samples was determined with a nano-drop spectrometer at 260 nm. 100 ng RNA were used in the reactions with the Invitrogen superscript III one-step RT-PCR kit. The PCR products were run on 3% ultrapure agarose with ethidium bromide at 95 V for 1 h. Gels were visualized on a UV light box.

Primers (all 5’ - 3’):

- ***Kif20b*** **exon 3–7:** FW:TGCTGAAAGACCCTCAAAGCATCCT, RV:ACTGGACTGGTCACAACTGTTCACG
- ***Kif20b*** **exon 17–19:** FW: GGTTCAGGCACTCAAGACATCAAGT, RV: CGATACTTCTTGCAGCAGTCTCCAT
- **Beta-actin (Actb gene):** FW: GATGACCCAGATCATGTTTGAGACC,
- RV: TAATCTCCTTCTGCATCCTGTCAGC

### Neuron electroporation

*The GFP*-*KIF20B* construct was electroporated into mouse cortical neurons. Shortly, neurons were dissociated from E14 mice cortices freed from meninges. After a cell count, the appropriate volume for one million neurons density was pipetted in eppendorf tubes and the neurons were spun down at 1.8 rpm for 10 min. The supernatant was carefully removed and the cells were re-suspended in 100 μl electroporation solution and 5 μg total plasmids were added (4 μg of *GFP*-*KIF20B* plasmid, 1 μg mCherry plasmid) and the suspension was mixed gently. The neurons were transferred carefully into an electroporation cuvette and electroporated with an Amaxa Electroporator following the instructions of the manufacturer for mouse cortical neurons (setting: O-005). Immediately after electroporation, 500 μl medium with serum was added to the cells and the suspension is carefully transferred into an eppendorf tube. 200 μl of cell suspension was then plated on either glass bottom dishes or coverslips and plating medium is added to make up 1 ml. Medium was replaced after 3 h with neuron growth medium with B27. Cells were grown at 37 °C, with 5% CO_2_ until usage.

### DiI tracing

Lipophilic DiI (1,1’-Dioctadecyl-3,3,3’,3’-Tetramethylindocarbocyanine Perchlorate) was used to stain neurons in E15.5 control and *Kif20b* mutant brains. Embryos were collected from pregnant heterozygote mothers at E15.5 and brains were dissected and fixed in 4% PFA for 2 days. A single small DiI crystal (Invitrogen, D-282) was then placed in the mid-lateral cortex of each hemisphere of each fixed brain using a needle pin. Brains were incubated at 37 °C in the dark for 3 days. Next, brains were cut in coronal sections at 100 μm on a Leica VT1000S vibratome and sections stained with DAPI (Invitrogen, D1306). Sections were mounted on slides with VectaShield (Vector Laboratories, H-1000) mounting medium. Z-stack images were taken of individual pyramidal neurons in the cortex at 40× on a Zeiss AxioImager.Z1 fluorescent microscope. Analysis of individual neurons was completed with ImageJ and NeuronJ by creating a maximum intensity projection of stacked images.

### Golgi staining

The Golgi method was used to stain neurons in E18.5 control and *Kif20b* mutant brains. Embryos were collected from pregnant heterozygote mothers at E18.5 and brains were dissected and rinsed with distilled water. The FD Rapid GolgiStain™ Kit (FD NeuroTechnologies, Inc., PK401) was used to stain whole brains, with the modification of placing brains in Solution A/B for 3 weeks instead of 2 at room temperature. Brains were next placed in Solution C for 1 week at 4 °C. Afterwards, brains were flash frozen on dry ice and stored at -80 °C until they were mounted in distilled water and cut on a Leica CM 3050S cryostat. Coronal sections were cut at 100 μm and mounted on slides with Solution C. The sections were allowed to dry for 3 days before completing the staining procedure with Solutions D and E as per instructions in the FD Rapid GolgiStain™ Kit. Slides were coverslipped with CytoSeal 60 (Thermo Scientific, 8310-4). Z-stack images were taken of individual pyramidal neurons in the cortex at 40× on a Zeiss AxioImager.Z1 microscope with Brightfield illumination. Neurons were also traced with Neurolucida Neuron Tracing Software (MBF Bioscience) for analysis. Analysis was completed by combining results from Neurolucida with results obtained from minimum intensity projection stacked images in ImageJ and NeuronJ.

### Neuron live imaging

The neurons were allowed to grow at 5% CO_2_ and 37 °C for 45 to 54 h in the incubator before imaging, and kept at 37 °C and 5% CO_2_ in the microscope controlled environmental chamber between time 0 and 6 h. The microscope was a Zeiss AxioObserver with an inverted 20× objective, a motorized stage, and Definite Focus that allowed for multiple scenes within each chamber to be imaged. The camera was AxioCam Mrm and image analysis was done with Zeiss Zen software.

## Results

### Loss of *Kif20b* disrupts morphogenesis of pyramidal neurons in embryonic cortex

**Fig. 1:**
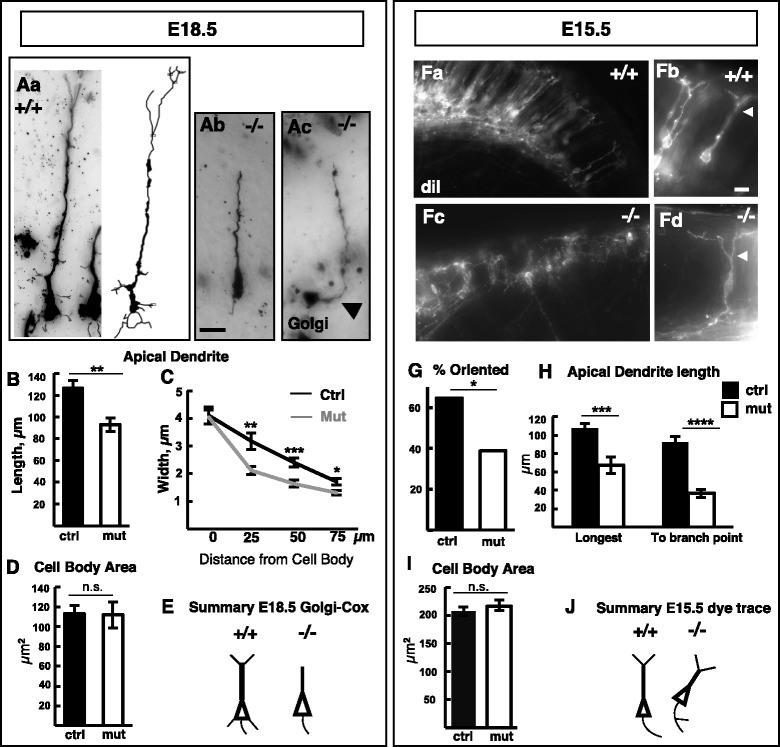
Cortical neurons show abnormal morphologies in *Kif20b*-/- brains. **A** Example images of individual pyramidal neurons labeled by Golgi-Cox staining in *Kif20b* control (+/+, a) and mutant (-/-, b and c) E18.5 brains. An example projection of 3D-tracing is shown of a control neuron (**a**). *Arrowhead* in (**c**) points to proximal apical dendrite. **B** Apical dendrites of mutant pyramidal neurons were shorter. **C** Mutant neurons’ apical dendrites tapered more quickly and remained thinner distally. **D** Average cell body area at E18.5 was the same in control and mutant brains. **E** Schematic summary of morphological differences of E18.5 mutant cortical neurons. **F** Examples of retrogradely labeled neurons in control (+/+, a and b) or *Kif20b* mutant (-/-, c and d) E15.5 cortical plate from lateral diI crystal placements. *Arrowheads* indicate first branch point of apical dendrite. **G** Fewer mutant neurons were properly oriented with apical dendrites within 10° of perpendicular to the pia. *n* = 31 cells each from 4 control and 3 mutant brains, *p* = .04, Chi-squared test with n-1 correction. **H** *Kif20b* mutant neurons had shorter apical dendrites and shorter distance to first branch point. Distance to first branch point was still significant after normalization to cortical thickness. **I** Average cell body area at E15.5 was the same in control and mutant brains. **J** Schematic summary of findings from diI retrograde tracing in E15.5 cortices. For E18.5, *n* = 26 control and 25 mutant neurons from 4 control (3 +/+, 1 +/-) and 3 mutant (-/-) brains. For E15.5, *n* = 30 control and 31 mutant neurons from 4 control (1 +/+, 3 +/-) and 3 mutant (-/-) brains. Error bars are s.e.m. *, *p* ≤.05, ** *p* ≤.01, *** *p* <0.001, **** *p* <0.0001; student’s *t*-test for all except panel G. Scale bars, 20 μm

We examined individual neocortical neuron morphologies of control and *Kif20b* mutant cortices using Golgi-Cox staining at E18.5 (Fig. 1A, a-c). Neonatal lethality precluded examination of fully developed neurons. Interestingly, *Kif20b* mutant neurons displayed several morphological differences compared to controls. The apical dendrites were ~ 30% shorter (Fig. 1B) and had fewer terminal branches detectable. Furthermore, fewer neurites near the soma were detected, and some neurons appeared misoriented. One mutant neuron appeared to lie on its side with the apical dendrite curving up toward the pia (Fig. 1Ac, arrowhead). The mutant neurons’ apical dendrites appeared thinner; measurements showed they had the same width at their base, but then tapered more quickly and were thinner than controls (Fig. 1C). To control for neuron size, and since *Drosophila pav* mutant neurons are abnormally large [16], we compared neuronal cell body sizes of control and *Kif20b* mutants. However, mutant neuron somas were the same size as controls (Fig. 1D). Axons were not measured since Golgi staining did not reliably label them.

To test whether the neurite and orientation defects were detectable from early growth stages, we examined cortical neurons of E15.5 brains. Golgi-Cox staining does not label cortical neurons at this age, so retrograde dye-tracing was employed. In both control and mutant cortices, lateral diI crystal placements could retrogradely label pyramidal neurons in the cortical plate, confirming that at least some mutant neurons had extended long axons (Fig. 1 F, a-d). However, the mutant cortical plate appeared disorganized, with fewer neurons labeled than in controls, and less than half of those oriented properly with apical dendrites perpendicular to the pial surface (12/31 mutant cells vs. 20/31 control cells), (Fig. 1Fa,c, 1G). Mutant apical dendrites were only two thirds the normal height (Fig. 1H). Strikingly, the mutant apical dendrites branched much closer to the cell body, at about one third the normal distance (Fig. 1Fb and d, arrowheads, and 1H). Axons are not expected to branch much at this early age, but branching on proximal axons appeared increased among mutant neurons, with 5/15 mutant neurons having at least one detectable branch, versus only 3/22 control neurons detected with one branch each. Again, the average soma size was indistinguishable in mutants and controls (Fig. 1I). Together, these analyses demonstrate that *Kif20b* is required for multiple aspects of normal neuron morphogenesis in the developing cortex, including polarity, branching, and dendrite width. The requirements for *Kif20b* could be cell-autonomous or non-autonomous. For example, the shortening of apical dendrites was proportional to the decrease in cortical thickness at both E15.5 and E18.5. By contrast, the shortened distance to the first branch point of the apical dendrites remained significant even after normalization to cortical thickness. Therefore, to sort out cell autonomous requirements for *Kif20b* in neuron growth from non-autonomous effects due to abnormal brain size and shape, we pursued further analyses on dissociated cortical neurons in vitro. This also enabled us to analyze more features of neuron shape.

### *Kif20b* is required for normal polarization in dissociated cortical neurons

**Fig. 2:**
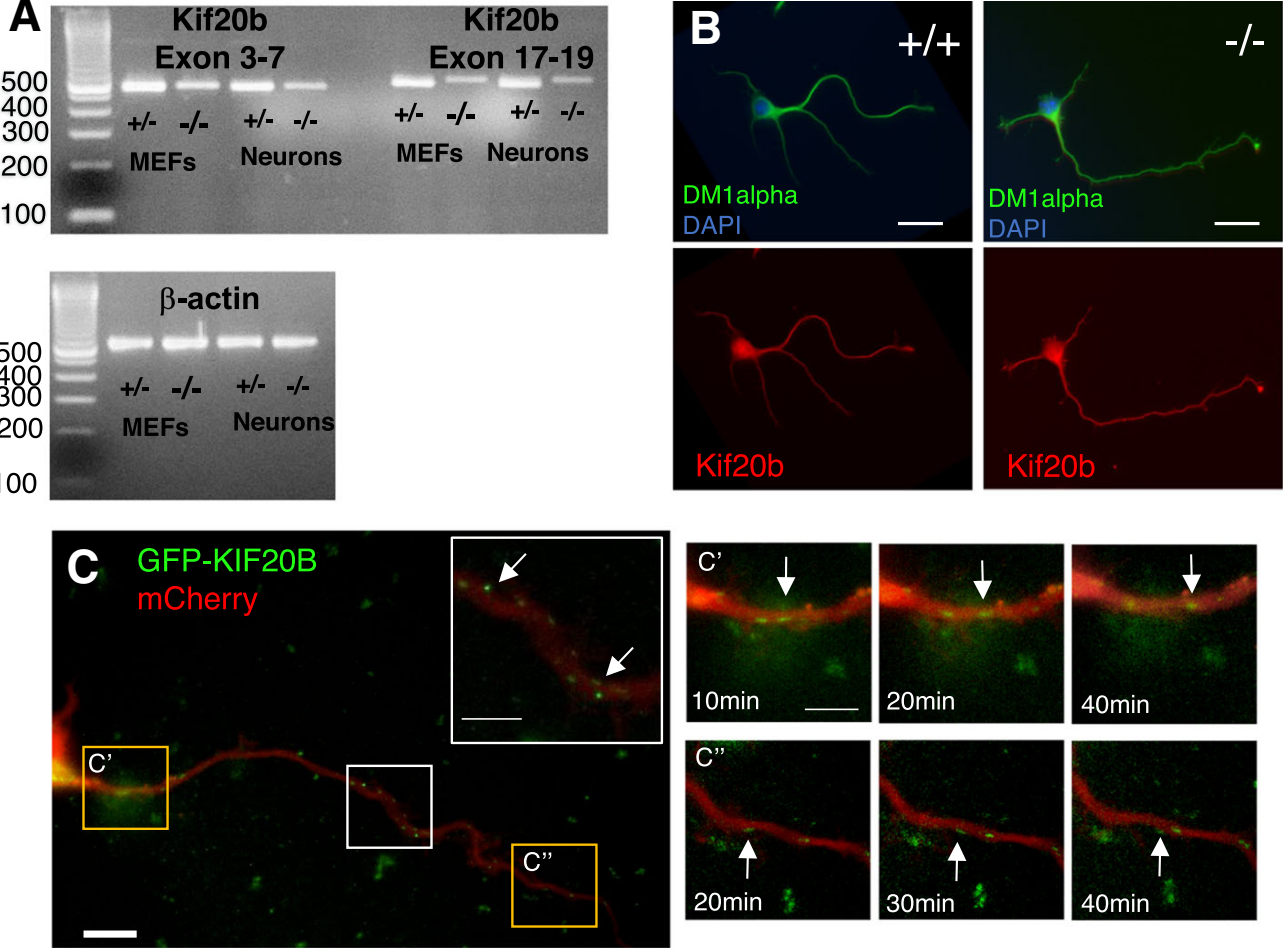
*Kif20b* expression and localization in cultured cortical neurons. **A** RT-PCR detects *Kif20b* weakly in neuron culture lysates (E14.5 plus 4DIV) after 30 cycles. Bands are stronger in MEFs. Bands are weaker in mutant samples due to the splice mutation [14]. **B** Immunocytochemistry for *Kif20b* does not detect signal in control neurons (+/+) above background seen in mutant (-/-) neurons. Images shown are of a polyclonal antibody to the full length protein [11], but are representative of staining appearance of five independent antibodies (see Methods). Scale bars, 20 μm. **C** Plasmids encoding GFP-tagged human KIF20B and mCherry were electroporated into mouse cortical neurons and imaged live. GFP puncta were distributed throughout the neurites and axon (*arrows*, inset). C’ and C”: Some GFP puncta moved anterogradely in the axon (*arrows* point to the same puncta at different time points). 10 μm scale bar and 5 μm in insets

To test for cell-autonomous roles of *Kif20b* in neuron morphogenesis, we first wanted to test expression of *Kif20b* in postmitotic neurons. Previously, we showed by in situ hybridization that *Kif20b* mRNA is expressed most strongly in the germinal zones of the embryonic brain and very weakly in neuronal layers. Also, Kif20b protein was readily detected in midbodies of dividing neural progenitors in control brains but undetectable in the *Kif20b* mutant progenitors [14]. Here, we first confirmed that *Kif20b* mRNA is expressed in postmitotic neurons by RT-PCR on cDNA from cortical neuron cultures (Fig. 2A). Beta-actin was used as a control for the amount of template cDNA. *Kif20b* amplicons could barely be detected after 25 cycles of PCR from neuronal cDNA, but were clearly detected from mouse embryonic fibroblasts (MEFs). After 30 cycles, *Kif20b* bands were clear in neuron samples, and stronger in MEFs. This indicates that *Kif20b* mRNA is present but not abundant in neurons. As seen previously, the bands were more weakly amplified from mutant samples, and higher molecular weight for exons 17–19 primers, due to the mutation causing aberrant mRNA splicing at the exon 18–19 junction [14]. By contrast, the Kif20b protein band could not be detected in immunoblots of the cultured neuron lysates. This is not surprising given that even in control whole brain lysates, the Kif20b protein band is very thin, denoting low abundance [14]. Next we tried immunocytochemistry to detect the localization of endogenous Kif20b protein in dissociated neurons. However, the diffuse signal detected throughout control neurons was also seen in mutant neurons, indicating that this is background (Fig. 2B). No specific signal was detected in control neurons over mutant neurons with any of several fixation conditions and independently-made polyclonal and monoclonal antibodies that were all verified for Kif20b reactivity ([11, 14, 15]; see Methods). To test where endogenous Kif20b protein would localize in neurons if we could detect it, we exogenously expressed GFP-tagged full-length human KIF20B, that localizes properly in dividing cells [11]. Though GFP-KIF20B overexpression caused death of many neurons, the small number of healthy neurons had numerous GFP puncta in the cell body, axons and minor neurites (Fig. 2C). Some of these GFP puncta were motile and moved anterogradely (Fig. 2C’ and 2C”). A previous study showed that a tagged KIF20B motor head domain acted as a translocating motor that in mature cultured hippocampal neurons tended to accumulate in axons [17]. Together these data suggest that embryonic cortical neurons express Kif20b at low abundance, that the motor distributes through immature neurites by translocating on microtubules, and can accumulate in axons as the neuron matures.

**Fig. 3:**
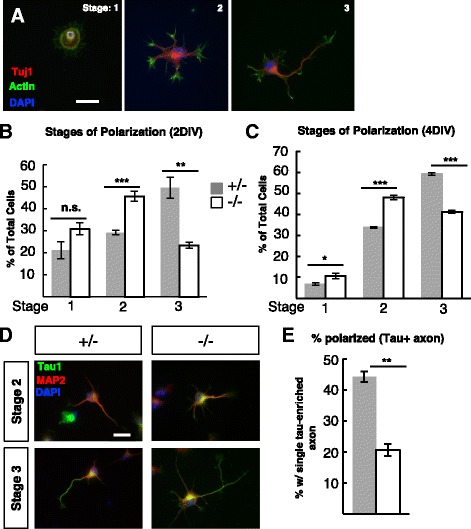
*Kif20b* mutant neurons are less polarized at 2 days or 4 days in vitro. **A** Representative images of dissociated E14.5 cortical neurons that are at Stage 1 (unpolarized), Stage 2 (multipolar), or Stage 3 (polarized; having one neurite at least twice as long as any other) after two days in vitro, and stained for neuron-specific beta-III tubulin (Tuj1, *red*), actin (phalloidin, *green*), and nuclei (DAPI, *blue*). **B** The average percentage (±s.e.m.) of neurons at each stage in cultures from control heterozygous (+/-, *gray bars*) or *Kif20b* mutant (-/-, *white bars*) cortices. Mutant cultures have fewer polarized neurons (stage 3) and more multipolar neurons (stage 2) compared to control heterozygous cultures. *n* = 6 control (+/-) and 6 mutant (-/-) coverslips from 3 independent experiments with 2258 total control and 1335 mutant cells scored. **C** After 4 days in vitro (4DIV), *Kif20b* mutant neurons are still less likely to be polarized. *n* = 4 control (+/-), *n* = 3 mutant (-/-) coverslips from 3 independent experiments; 827 control, 510 mutant neurons. **D** Representative images of dissociated cortical neurons stained for MAP2 and Tau1. Polarized neurons have a single, tau1-enriched neurite that is the axon. **E** The average percentage (±s.e.m.) of neurons with a single tau-enriched neurite (polarized) is decreased in *Kif20b* mutant cultures after 2 DIV. *n* = 3 control (+/-), 3 mutant (-/-) coverslips from 3 independent experiments; 615 control, 381 mutant neurons. *, *p* <0.05; **, *p* <0.001; ***, *p* <0.0001; n.s., not significant, *t*-test. Scale bars, 20 μm

Next we tested whether *Kif20b* is required for normal polarization of isolated cortical neurons in vitro. When embryonic cortical pyramidal neurons are dissociated and then cultured, they re-establish polarity by progressing through defined stages (Fig. 3A). We observed that after two days in vitro (DIV), in control cultures 50% of the neurons were polarized (stage 3, with one neurite at least twice as long as the next longest neurite); but in *Kif20b* mutant cultures, only 23% were polarized (Fig. 3B). Furthermore, 29% of control neurons were multipolar (stage 2), compared to 46% of mutant neurons. These data suggest that loss of Kif20b disrupts the progression from multipolar to polarized. To test whether polarization was simply delayed, we examined cultures at 4 DIV. While both controls and *Kif20b* mutant cultures had more polarized neurons at 4 DIV, the mutants still had a significantly smaller proportion polarized than controls (Fig. 3C). Finally, we confirmed that the *Kif20b* mutant neurons had a defect in axon specification and not simply axon growth by staining for the axon-enriched microtubule associated protein, tau1. Indeed, only about half as many mutant neurons had specified an axon as indicated by tau1-enrichment as controls did, confirming a polarization defect (Fig. 3D, E). No cells with extra axons were observed. This robust polarization defect could be an exacerbated version of the disorganization and mis-orientation phenotype seen in vivo at E15.5 (Fig. 1Fc, G).

### Shootin1 is less enriched in growth cones of *Kif20b* mutant axons

**Fig. 4:**
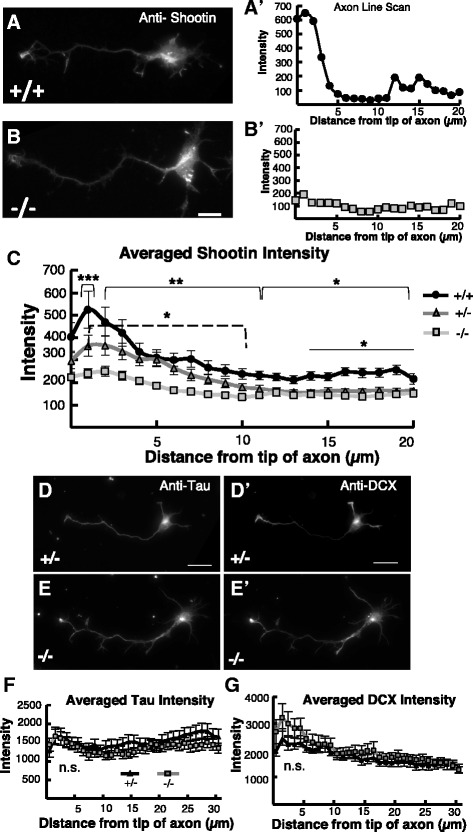
*Kif20b* mutant axons have reduced enrichment of Shootin1 in the growth cone. **A** Immunostaining for endogenous Shootin1 with anti-Shootin1 antibody [15] shows Shootin1 enriched in the axonal growth cone of a control (+/+) Stage 3 neuron. ***A’*** Linescan of Shootin1 staining intensity from image in **A**, starting from the tip of the axon and extending 20 μm shows a peak at the growth cone that flattens in the axon shaft. **B** Shootin1 immunostaining reveals Shootin1 in the soma of a polarized *Kif20b* mutant (-/-) neuron, but little enrichment in the distal axon. ***B’*** Linescan of Shootin1 staining intensity from image in **b** shows a severely reduced peak of Shootin1 at the tip of the mutant axon. **C** Averaged line scans of anti-Shootin1 signal intensity of 78 +/+, 101 +/-, and 100 -/- axons from 3 independent experiments show significantly bigger peaks in control axons than mutants. (+/+, *black circles*; +/-, *gray triangles*; -/-, *light gray squares*). *, *p* <0.05; **, *p* <0.01; *** *p* <0.001, *t*-test. Solid brackets compare wild-type (+/+) with mutant (-/-) for all points under each bracket. *Dashed bracket* compares heterozygous controls (+/-) with mutant (-/-) for all points under the bracket. Line compares +/+ with +/- for all points under the line. **D**, **E**, and **F** Tau immunostaining and axonal linescans reveal no significant difference in distribution or intensity between control and mutant neurons. ***D’***, ***E’***, and ***G***. DCX (doublecortin) immunostaining and axonal linescans show similar high distal distributions and intensities in control and mutant neurons. *n* = 40 +/- and 40 -/- cells for both Tau and DCX linescans from two independent culture experiments (2 animals each, 2 coverslips from each animal). Scale bar = 10 μm for **A** and **B**. Scale bar =20 μm for **D** and **E** n.s., not significant, *t*-test

The reduced ability of *Kif20b* mutant neurons to form an axon in vitro could be due to defective localization of a relevant cargo or binding partner. We hypothesized that the *Kif20b* polarization defect could be at least partly explained by a change in Shootin1 localization, based on previously published data. Shootin1 was shown to localize to axonal growth cones coincident with polarization, and afterward during rapid axon growth. Depletion of Shootin1 disrupted neuron polarization and axon extension in vitro [18, 19]. In addition, Shootin1 was found to immunoprecipitate with Kif20b from embryonic mouse brain lysates and human cell lines [15, 20]. Further, Kif20b knockdown caused de-localization of mCherry-Shootin1 [15]. To test whether genetic loss of *Kif20b* alters endogenous Shootin1 distribution in cortical neurons’ axons, we compared the axonal distribution of Shootin1 in *Kif20b* mutant and control axons by measuring the intensity of anti-Shootin1 immunostaining from the tip of the growth cone into the axon shaft. All axons that were analyzed had similar length to control that as a potentially confounding factor. As expected in control polarized neurons, Shootin1 immunostaining showed enrichment in the axonal growth cones compared to the axon shaft, with a peak of signal in the growth cone (Fig. 4A, A’). Interestingly, in *Kif20b* mutant polarized neurons, the Shootin1 signal was much weaker in the axonal growth cone (Fig. 4B, B’). Averaging axonal line scans over many cells confirmed that the *Kif20b* mutant axons had a significant reduction in Shootin1 accumulation in the growth cone and distal axon compared to control axons (Fig. 4C). Interestingly, there was even a slight difference in Shootin1 intensity between heterozygous (+/-) and wild-type (+/+) control axons farther from the growth cone, suggesting a dosage effect of Kif20b on the amount of Shootin1 in the axon tip. Furthermore, a small amount of Shootin1 enrichment in the growth cone above the axonal level was still seen in *Kif20b* mutant cells, suggesting that another mechanism can still enrich it at the growth cone. To determine if the mislocalization was specific to Shootin1, we examined two other axonal proteins, Tau and DCX (doublecortin). Tau immunostaining had similar intensity throughout the axon in both control and mutant axons (Fig. 4D, E, F). DCX immunostaining showed a normal high-distal localization [21] with similar intensity in the axons of both control and mutant neurons (Fig. 4D’, E’, G). Thus *Kif20b* mutant neurons can localize DCX and Tau properly to the developing axon, but not Shootin1. These data are consistent with previously published work suggesting that Kif20b influences distribution of exogenous tagged Shootin1 in the axon [15] but also that a myosin-based mechanism localizes Shootin [18]. Thus, the reduction of Shootin1 enrichment in *Kif20b* mutant axonal growth cones may at least partly explain the neuron polarization defect.

### Polarization and structure differences are not due to a shift in cell or layer fates

**Fig. 5:**
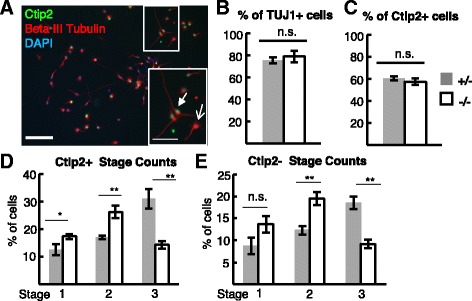
Morphological changes in *Kif20b* mutant neurons are not due to cell type changes. **A** Representative field image of E14.5 cortical cells cultured for 2 days in vitro, immunostained for neuronal marker beta-III-tubulin (Tuj1, *red*), layer 5/6 marker Ctip2 (*green*) and nuclei (DAPI, *blue*). Filled arrow points to Ctip2^+^ neuron; open arrow to Ctip2^-^ neuron. Scale bars, 100 μm and 10 μm in inset. **B** The percentage of cells in control (+/-, *gray bar*) and mutant (-/-, *white bar*) cortical cultures expressing neuronal marker (TuJ1^+^) is not significantly different. *n* = 3 coverslips each with 1120 control cells and 769 mutant cells analyzed. **C** The percentage of neurons marked by Ctip2 does not differ in control and mutant cultures. **D** and **E** The average percentage (±s.e.m.) of Ctip2^+^ or Ctip2^-^ neurons at each stage in cultures from control heterozygous (+/-, *gray bars*) or *Kif20b* mutant (-/-, *white bars*) cortices. Mutant cultures have fewer polarized neurons (stage 3) and more multipolar neurons (stage 2) compared to control heterozygous cultures. For C, D, and E, *n* = 6 control (+/-) and 6 mutant (-/-) coverslips (2258 and 1335 neurons total, respectively). Error bars are ± s.e.m; *, *p* <0.05; **, *p* <0.01, n.s., not significant, *t*-test

It was possible that the polarization defect and reduction in Shootin1 localization could be due to cell fate changes in the *Kif20b* mutant cultures. Indeed, cytokinesis mechanisms have been shown to play roles in daughter cell fate determination [22–24], and we had previously demonstrated cytokinesis defects in the *Kif20b-/-* embryonic cortex [14]. To rule out fate change as a cause of polarity loss, we compared the percentages of neurons (Tuj1^+^) and of layer 5/6 neurons (Ctip2^+^) in control and mutant cultures (Fig. 5A). Both the percentage of neurons and of Ctip2^+^ neurons were not different (Fig. 5B, C), indicating that the mix of cell types or neuronal layer types is not altered in *Kif20b* mutant cultures. To further control for this, Ctip2^+^ and Ctip2^-^ neurons were directly compared for polarization in control and mutant cultures. Both showed the same robust polarization defect (Fig. 5D, E). These data strongly argue that *Kif20b* influences neuronal polarization and morphological development through a mechanism independent of cell fate and required by both deep and upper layer types.

### *Kif20b* mutant neurons that do polarize have structural changes in both axons and minor neurites

**Fig. 6:**
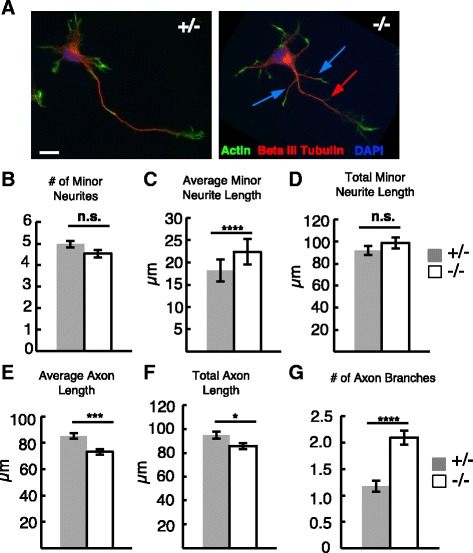
*Kif20b* mutant neurons that polarize have shorter axons, longer minor neurites, and more axon branches. **A** Representative images of control (+/-) and mutant (-/-) polarized (Stage 3) neurons from E14.5 cortical cultures after 2 DIV, stained with phalloidin (actin, *green*), anti-beta-III-tubulin (*red*), and DAPI (*blue*). *Blue arrows* indicate axon branches. *Red arrow* indicates branch with only actin. Scale bar, 10 μm. **B** The average number of minor neurites (nascent dendrites) was slightly decreased in *Kif20b* mutant neurons (-/-, *white bars*), but was not statistically significant. **C** Individual minor neurites of *Kif20b* mutant neurons (-/-, *white bars*) are longer on average than controls’ at 2DIV*. n* = 323 +/-, 251 -/- minor neurites. **D** Summed dendritic length per cell is slightly increased in mutant neurons but is not statistically significant due to variable minor neurite number. **E** *Kif20b* mutant neurons (-/-, *white bars*) have shorter axons. *n* = 204 +/- and 260 -/- neurons. **F** Total axonal length including side branches was shorter in mutant neurons than controls. n = 202 +/- and 260 -/- neurons. **G** The average number of axon branches (containing tubulin) on mutant neurons was increased compared to controls. All length measurements were made based on tubulin staining not actin signal. For **B**, **D** and **G**, *n* = 79 +/-, 65 -/- neurons. Error bars are ± s.e.m. *, *p* <0.05; ***, *p* <0.001; ****, *p* <10^-6^; n.s., not significant

We hypothesized that if *Kif20b* helps establish polarity, then the mutant neurons that did polarize might have “weak” polarity, i.e., axons that are more dendrite-like (shorter and wider with more branches) and minor neurites (nascent dendrites) that are more axon-like (longer and thinner with fewer branches). Some observations are consistent with this idea (Fig. 6A-G). First, the minor neurites of *Kif20b* mutant polarized neurons averaged 29% longer than controls, and the average number of minor neurites per cell was not different (Fig. 6B, C). Furthermore, mutant axons were 14% shorter, but surprisingly had nearly twice as many collateral branches as control axons (Fig. 6E, F, G). Thus, loss of Kif20b appears to cause minor neurites to be longer (more like axons) and axons to be shorter and more branched (more like dendrites). A previous study had found that RNAi of *Kif20b* in cultured hippocampal neurons caused reduced axon length, but did not note changes in dendrites or axon branching [15].

**Fig. 7:**
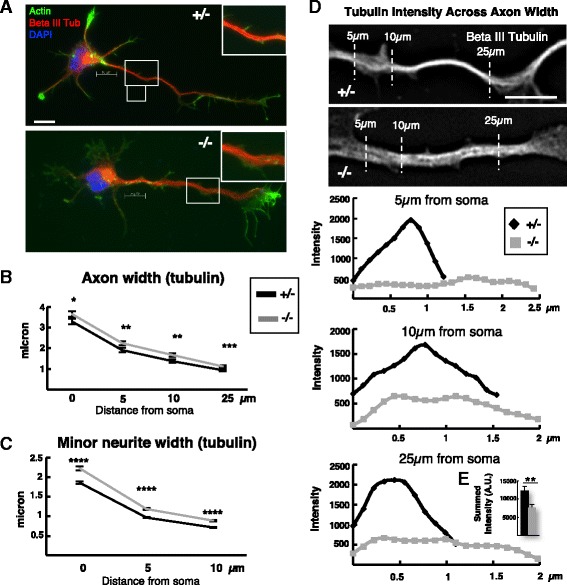
*Kif20b* mutant neurons have wider axons and minor neurites than controls. **A** Representative images of polarized control (+/-) and mutant (-/-) neurons cultured for two days and stained for actin (*green*), beta-III-tubulin (*red*) and nuclei (DAPI, *blue*). Insets show magnified axon segments for comparison. **B** Average width (±s.e.m.) of mutant axons (-/-, *gray line*) is greater than controls (+/-, *black line*) at each given distance from the edge of the soma. *n* = 79 +/- and 65 -/- axons. **C** Average width (±s.e.m.) of mutant minor neurites (*gray line*) is increased over controls (*black line*) at each given distance from the cell body. *n* = 323 +/- and 262 -/- minor neurites. **D** Representative images of and linescans across control (+/-) and mutant (+/-) axons stained for beta-III tubulin show that tubulin intensity was lower, more irregular, and more spread out in mutants than controls. Dotted lines represent where tubulin intensity linescans were taken. **E** Inset bar graph shows average total tubulin intensity summed across the width 25 μm from the soma was significantly decreased in mutant axons compared to controls. *n* = 15 each +/- and -/- from 1 experiment. *, *p* <.05; **, *p* <.01; ***, *p* <.001; ****, *p* <10^-9^. 10 μm scale bars

Next we tested whether the weakened polarity characteristics of *Kif20b* mutant neurons alter axon and minor neurite width. In a mature pyramidal neuron, the axon is long and thin with constant caliber, while dendrites are shorter, wider, and more tapered. We had observed in vivo that *Kif20b* mutant pyramidal neurons had thinner apical dendrites (Fig. 1C). Also, RNAi of the different Kinesin-6 family member Kif23 in cultured sympathetic neurons had also caused dendrite thinning [7]. Therefore we measured the widths of axons and minor neurites at given distances from the cell body, using neuronal (beta-III) tubulin staining (Fig. 7A). Surprisingly, in culture, both the axons and minor neurites of *Kif20b* mutant neurons were significantly wider than controls (Fig. 7A-C). At 10 microns from the soma, mutant axons averaged 21% wider than controls and mutant minor neurites were 24% wider. The increased widths appear to correlate with looser microtubule packing (Fig. 7A, insets). To confirm this, the tubulin intensity was measured by linescans across the widths of the axons at 5 μm, 10 μm, and 25 μm from the cell body. Interestingly, while control axons had compact tubulin intensity distributions with a clear peak, mutant axons had wider tubulin distributions with lower, irregular peaks (Fig. 7D). Furthermore, the average total tubulin intensity at 25 μm from the soma is significantly decreased in the mutant axons, suggesting that not only do microtubule bundles have more spaces between them, but also that there is less tubulin in the axons at a given distance (Fig. 7E). These data show that Kif20b not only supports axon specification in the cue-free in vitro environment, but also helps set up the structures of nascent axons and dendrites, possibly by regulating microtubule packing. Indeed, previous in vitro work showed that adding KIF20B protein to microtubules was sufficient to cause them to become crosslinked and bundled in an ATP-dependent manner [11].

To confirm that the changes in branching and neurite width were not due to a change in neuron types in the Kif20b mutant cultures, we again controlled for this. Indeed, the foregoing findings held true when we controlled for neuron layer type: *Kif20b* mutant Ctip2^+^ neurons had increased axon branching, minor neurite length, and neurite width, compared to Ctip2^+^ control neurons (Additional file 1: Figure S1). Worth noting, Ctip2^+^ neurons did not differ significantly from Ctip2^-^ neurons by our measurements, and were affected similarly by Kif20b loss. This suggests that at least at early days in vitro, deep and upper layer pyramidal neurons develop similarly and both require Kif20b.

### *Kif20b* mutant neurons have longer filopodia with increased microtubule invasion

**Fig. 8:**
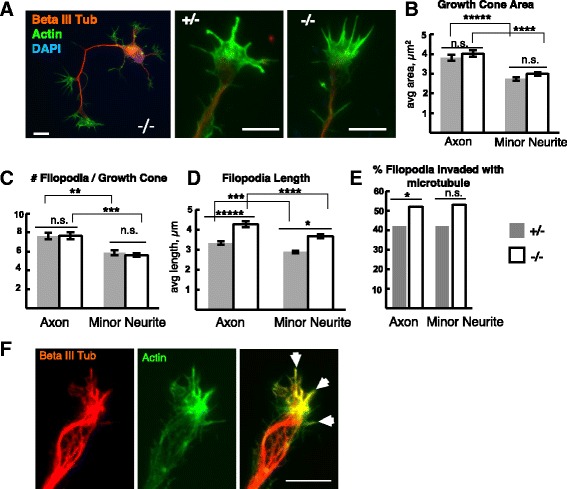
*Kif20b* mutant neurons’ growth cones have longer filopodia. **A** Representative images of control and *Kif20b* mutant growth cones of dissociated E14.5 cortical neurons cultured 2 DIV and immunostained to label neuronal tubulin (TuJ1, *red*), actin (phalloidin, *green*), and nuclei (DAPI, *blue*). Scale bars 10 μm. **B** The growth cones of axons are significantly larger in area than those of minor neurites, but similar in control (+/-, *gray bars*) and mutant (-/-, *white bars*) cells. **C** The average number of filopodia per growth cone did not differ significantly in *Kif20b* mutant neurons on either axons or minor neurites. For B-C, *n* = 38 control (+/-) axonal and 54 minor neurite growth cones; mutant (-/-) *n* = 37 axonal and 49 nascent dendritic growth cones. **D** *Kif20b* mutant neurons have longer filopodia on both axonal and nascent dendritic growth cones. Axonal filopodia are slightly but significantly longer than dendritic filopodia regardless of genotype. *n* = 217 control and 308 mutant filopodia from 39 and 37 axonal growth cones, and *n* = 155 +/- and 134 -/- filopodia from 25 and 22 minor neurite growth cones respectively. For (**A**-**D**), error bars are ± s.e.m. *, *p* < 0.01; **, *p* <10^-3^; ***p <10^-5^; ****p <10^-6^; *****, *p* <10^-8^; n.s., not significant, *t*-test. **E** In *Kif20b* mutant neurons, a higher percentage of growth cone filopodia contain detectable microtubules than in controls, in both axons and minor neurites. *n* = 217 for +/- and 308 -/- filopodia on axonal growth cones and n =155 +/- and 134 -/- filopodia on minor neurite growth cones. *, *p* <0.05 Fisher’s exact test. **F** Axonal growth cone labeled for actin (*green*) and tubulin (*red*). Arrowheads mark filopodia containing microtubules

To address the possible mechanisms for increased minor neurite length and axon branching in *Kif20b* mutant neurons, we examined the structures of growth cones on both axons and nascent dendrites of polarized (Stage 3) neurons. Axons, axon branches, and minor neurites can all be tipped by growth cones, and their size and morphology varies (Fig. 8A). There was no significant difference between control and *Kif20b* mutant growth cone areas, though axonal growth cones were at least a third larger than minor neurite growth cones in both controls and mutants (Fig. 8B). *Kif20b* mutant growth cones also did not differ in the number of filopodia per growth cone, and had about 35% more filopodia on axonal than minor neurite growth cones, proportional to the larger area, just like controls (Fig. 8C). Surprisingly however, *Kif20b* mutant filopodia were about 30% longer than control filopodia on both axons and minor neurites, with axonal growth cones having longer filopodia than minor neurite growth cones in both cases (Fig. 8D).

All filopodia contain bundled actin, but when microtubules penetrate filopodia this may lead to stabilization of the filopodium, inducing a branch or growth cone extension [25, 26]. We hypothesized that longer filopodia could be due to increased microtubule invasion. We tested this by comparing the percentage of filopodia with detectable tubulin in control and mutant polarized neurons. Consistent with our hypothesis, the filopodia on *Kif20b* mutant growth cones were more likely to contain tubulin than control filopodia, suggesting increased microtubule invasion of the growth cone periphery (Fig. 8E, F). Together with the above analyses, these data suggest that loss of *Kif20b* only partly blocks polarization, and the cells that do polarize have structural changes in both axons and minor neurites - length, branching, width, and filopodia length – suggestive of dysregulated microtubule packing or microtubule-actin interactions.

### *Kif20b* mutant axons retract more and pause less than control axons

**Fig. 9:**
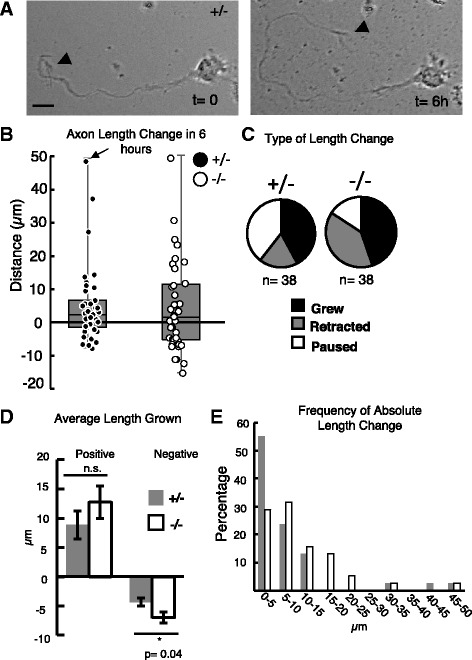
*Kif20b* mutant axons are more likely to retract and retract farther than controls. **A** Example brightfield images of live 2 DIV cortical neuron imaged at time 0 h and 6 h. This neuron grew its axon 48 μm. *Arrowheads* point to growth cone. Scale bar, 10um. **B** Change in axon length after 6 h for each individual neuron was plotted as a scatter plot, and the distribution as a box plot. The dot representing the axon imaged in A is marked by an arrow. *n* = 38 control (+/-, *black dots*) and 38 *Kif20b* mutant (-/-, *white dots*) neurons, imaged over 7 different experiments from 6 +/- embryos and 7 -/- embryos. Axons of similar starting lengths were chosen for imaging (averages 52 and 48 μm for +/- and -/-, respectively, *p*-value n.s.). **C** Mutant axons (-/-) were more likely to retract and less likely to pause than control axons (+/-) in 6 h. The change in category distribution is significantly different by Fisher’s exact test, *p* = 0.03. “Grew” signifies at least +3 μm length change; “Retracted”, at least -3 μm; “Paused”, within ±2.9 μm of starting length. **D** The average length retracted in 6 h among axons that shrank was significantly increased in the *Kif20b-/-* neurons. The average length increase in 6 h among axons that grew was not significantly different. Here, all above-zero changes were averaged for “positive” and all below-zero changes were averaged for “negative”. **E** The absolute values of length changes were binned and plotted as a histogram to show greater length changes in *Kif20b* mutant axons (*white bars*) compared to heterozygous controls (*black bars*). The median was higher in the mutants (6.9 μm versus 4.4 μm, Mann-Whitney *U*-test *p* = 0.04). *, *p* <0.05; n.s., not significant, *t*-test

The preceding experiments showed that dissociated cortical neurons from *Kif20b* mutant brains are less likely to have polarized after a few days in vitro, and if polarized, have shorter and more branched axons with less compact tubulin. This suggests the mutant neurons are ineffective at stabilizing the axonal cytoskeleton to sustain consistent and rapid axon growth. To test this idea, we compared axon growth of live control and *Kif20b* mutant neurons in a 6 h period after 2 DIV (Fig. 9). As expected, the change in axon length after 6 h varied widely, with some axons having grown and some having retracted, ranging from +48 μm to -15 μm change in length (Fig. 9B). To break this down, we categorized each axon as “grew” (length change at least +3 μm), “retracted” (at least -3 μm), or “paused” (within +2.9 μm). Interestingly, in 6 h, more mutant neurons retracted and fewer paused compared to controls, while about the same number grew (Fig. 9C, Fisher’s exact test, *p* = 0.03). Of the axons with positive growth, the average length increase was not significantly different (Fig. 9D). However, of the axons that shrank, the average length decrease was significantly greater in the *Kif20b* mutant neurons compared to controls (Fig. 9D). Since the distribution of length changes was wide, and our categorization was arbitrary, we analyzed the data in one additional way to confirm that mutant axons behaved differently. Taking the absolute values of length changes and plotting the distribution shows a shift to greater length changes in the mutants (Fig. 9E). Together, these analyses suggest that the mutant axons have less consistent growth due to greater variability of length change. Loss of Kif20b makes axons more likely to retract, and if they do, to retract more. Kif20b’s functions may normally act as a brake on axon retraction.

## Discussion

The dramatic reorganization of the neuronal cytoskeleton during polarization and axon growth is an area of intense research. Recent work points to microtubule associated proteins being important downstream effectors of signaling cues that mediate neuronal polarization, axon guidance and branching [4]. While many kinesins have been shown to be important for intracellular transport in mature neurons, less is known about which kinesins are important for these early processes. Recent work in *Drosophila* or by mammalian knockdown approaches has suggested that mitotic kinesins are re-used in postmitotic neurons to organize microtubule arrays [7, 9].

**Fig. 10:**
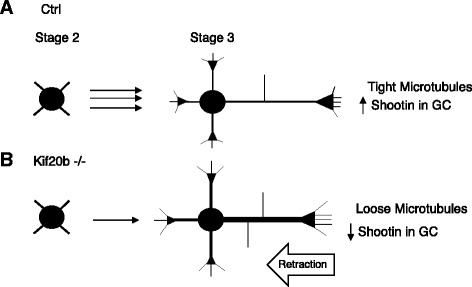
Summary of phenotypes of *Kif20b* mutant neurons seen in culture. **A** Normal cortical neurons transition from multipolar (Stage 2) to polarized with a single axon (Stage 3) properly when Kif20b is present. Kif20b may organize or stabilize microtubule arrays in the axon and growth cone, keeping the axon thin and limiting its branching, and preventing microtubule invasion of filopodia. Kif20b may also tether Shootin1 at the microtubule plus ends in the axonal growth cone, which reinforces axonal growth. Kif20b may thereby enhance stability and inhibit axon retraction. **B** When Kif20b is absent, some neurons fail to transition from stage 2 to stage 3. Those that do polarize show a variety of phenotypes: longer minor neurites, shorter axons with extra branches, wider axons and minor neurites, and longer filopodia. Microtubules appear more loosely organized and extend into more filopodia. Shootin1 enrichment in the growth cone is severely reduced

We report here for the first time through a specific genetic mutation that a Kinesin-6 family member, Kif20b, is required for neuronal morphogenesis in the mammalian brain. By in vivo and in vitro analyses, we find that loss of *Kif20b* disrupts development of cortical neurons, affecting polarization as well as neurite outgrowth, branching and caliber (see Fig. 10). The morphological changes are not due to cell fate changes, and are seen in different layer types. Our results suggest that Kif20b acts to stabilize or bundle microtubules in neurites to allow polarization, sustain axon growth, keep neurites thin, and minimize branching. Kif20b may also tether Shootin1 in the growth cone, which helps to maintain the axon in a growth state. Analysis of live neurons suggests that Kif20b may normally act to limit axon retraction. Thus, Kif20b helps to organize microtubule arrays in neurites to shape the neuron, and can also act by localizing effector molecule partners.

Our analyses highlight the importance of studying neuronal morphogenesis both in vivo and in vitro, because phenotypes vary and provide different clues to gene function. While the *Kif20b* mutant neurons had a robust polarization defect in vitro, clearly most neurons in vivo polarized and made an axon. A neuron polarizing in vitro has perhaps a more difficult task than one polarizing in vivo because there are no directional cues, and the substrate is stiff and two-dimensional. However, the orientation defect in the *Kif20b* mutant brains may reflect polarization defects in a subset of cells. Axon branching defects were seen both in vivo and in vitro, as well as effects on neurite width. In vitro we were able to analyze microstructures such as filopodia number and length. Previously, we reported that in the *Kif20b* mutant mouse brains, a subset of thalamocortical axons showed an axon guidance defect [13]. Though the exact cause of the misrouting is still unclear, our new data analyzing individual neurons suggest that axon guidance defects could be secondary to axon outgrowth timing or abnormal branching.

The Kinesin-6 gene family appears to have expanded by duplication during evolution [6, 27]. *C. elegans* has one Kinesin-6 gene, *zen-4*, orthologous to *Kif23*/MKLP1. *Drosophila* has two Kinesin-6 genes, *pavarotti* and *subito*. It may be that three members of this family are needed to build a more complex nervous system or larger neurons. While gene redundancy is often observed in neural development to ensure robustness [3], this appears not to be the case for the mammalian Kinesin-6 family, since loss of *Kif20b* causes deleterious phenotypes in both cytokinesis and neuron development that are not compensated for by *Kif23* or *Kif20a* ([14]; this work). Kif20b must have some molecular function that the other two cannot substitute for. The family members are highly homologous in the motor domain, but the stalks and tails are completely divergent, enabling interactions with distinct binding partners and cargoes. The significance of the extra- long stalk of Kif20b is not known, but it may increase flexibility, or allow the motor to bind microtubules or other proteins at a greater distance apart.

This work substantially deepens the evidence that Kinesin-6 family members function in neuronal development. Previous RNAi data supported roles in mammalian neurons for Kif23/MKLP1 in microtubule organization and neurite growth [7]. Knockdown or mutation of the *Drosophila Kif23* ortholog *Pav* caused increased microtubule sliding and excess axon branching [9]. Kif20b knockdown was shown to decrease axon length and Shootin1 distribution [15]. Here we provide genetic evidence for a requirement for *Kif20b* in multiple aspects of mammalian neuron morphogenesis. Kif20b loss has morphological consequences throughout the cell, consistent with the appearance of exogenous GFP-KIF20B throughout the neurites. As the cell matures, Kif20b may accumulate at the axon tip due to axonal preference of the motor head as shown in [17]. The appearance of looser microtubules in neurites and more microtubules in growth cone filopodia suggests Kif20b functions in microtubule crosslinking or packing organization in neurites. Indeed, the wider neurites are reminiscent of the wider midbodies we previously observed in dividing neural progenitors [14]. Both these phenotypes could be explained by loss of microtubule bundling activity of Kif20b that was demonstrated in biochemical assays [11]. A key process driving neuron polarization is likely to be local stabilization and bundling of parallel microtubules [4, 28]. As the growth cone advances, microtubule bundling at the neck may be a crucial requirement for converting the growth cone into axonal structure [29]. Thus, defective microtubule bundling could explain the mutant cells’ difficulty forming the axon, regulating neurite width, and preventing axon retraction.

Interestingly, filopodia length and axon branching are linked mechanistically by microtubule invasion. This work provides one of the few known manipulations to increase the length of growth cone filopodia or increase microtubule penetration into them [25, 26]. Axonal branches are initiated as filopodia [30] and are dependent on filopodia formation [31]. Formation of axon branches correlates with localized splaying and breaking of microtubules [2, 26]. Thus, the increased axon branching and longer filopodia in *Kif20b* mutants could both be related to a loss of microtubule crosslinking or tight bundling.

Kif20b is likely to have functions beyond microtubule organization. For example, as a translocating motor protein, it can transport proteins or link them to microtubules. We have provided evidence that at least one endogenous polarity protein, Shootin1, requires Kif20b for enriched localization at the axon growth cone. The role of Shootin1 is proposed to reinforce the identity of the axon and keep it growing [18, 19]. Kif20b may transport Shootin1 from the cell body to the growth cone, or it may help retain it at the growth cone after transport by other mechanisms [18]. The high abundance of Shootin1 relative to Kif20b, as well as the remaining weak Shootin1 signal in *Kif20b* mutant axons support the notion of another transport mechanism. Shootin1 has also been shown to bind the actin cytoskeleton and mediate traction of the growth cone [19]. Thus through Shootin1, Kif20b might link microtubules and actin. Importantly, there may not be one Kif20b function that explains all the phenotypes. There may well be as yet unidentified binding partners that Kif20b transports or tethers at different locations or times in development.

## Conclusions

These analyses considerably advance our knowledge of post-mitotic roles of Kif20b in neuron polarization, branch inhibition, and axon growth. Kif20b may affect these processes by its activities in organizing microtubules and localizing effector proteins. Loss of Kif20b appears to have different consequences for mammalian neuron morphology than loss of the other Kinesin-6 family member Kif23. Understanding how different motor proteins and other microtubule associated proteins enable neurons to reorganize their cytoskeletons during early polarization, axon growth, and arborization is fundamental to knowing how the brain is wired and changes during learning. In addition, there are important implications for human health. Since the *Kif20b* mouse mutant is a novel model for human microcephaly, it is important to know that there could be defects in connectivity in addition to the small brain size in some cases of microcephaly. Finally, *KIF20B* is elevated in several cancers [6, 32, 33], and while that could be due to its role in cell division, our data suggest it could also be due to effects on cell motility and morphology that could enhance metastasis.

## Additional file

Additional file 1: Figure S1. Morphological abnormalities in *Kif20b* mutant cells are observed in both Ctip2^+^ and Ctip2^-^ neurons. A. The average number of minor neurites is not different in *Kif20b* mutant cells (white bars) whether Ctip2^-^ or Ctip2^+^. B. Minor neurites are longer on average in *Kif20b* mutant neurons (white bars) than in control neurons (gray bars), whether Ctip2^+^ or Ctip2^-^. C. *Kif20b* mutant axons (white bars) have more branches than control neurons (gray bars) whether Ctip2^+^ or Ctip2^-^. D. Axons and minor neurites of *Kif20b* mutant neurons are wider on average than controls, regardless of whether they are Ctip2^+^ or not. Measurements done using images of tubulin immunostaining at same exposure times. Ctip2^+^ neurons n = 43 +/- and 40 -/-. Ctip2^-^ neurons *n* = 36 +/- and 25 -/-. Error bars are + s.e.m; *, *p* <0.05; **, *p* <0.01, ***, *p* <0.001, **** *p* <10^-5^, ***** *p* <10^-7^; n.s., not significant, *t*-test. (PDF 32 kb)

## Abbreviations

DIV: Days in vitro
E: Embryonic day
ENU: N-ethyl-N-nitrosourea
