# Supplementary figures and images for: Mutation of Kinesin-6 Kif20b causes defects in cortical neuron polarization and morphogenesis
Source: Neural Dev. 2017 Mar 31;12:5. doi: 10.1186/s13064-017-0082-5 (PMC5374676; doi:10.1186/s13064-017-0082-5)

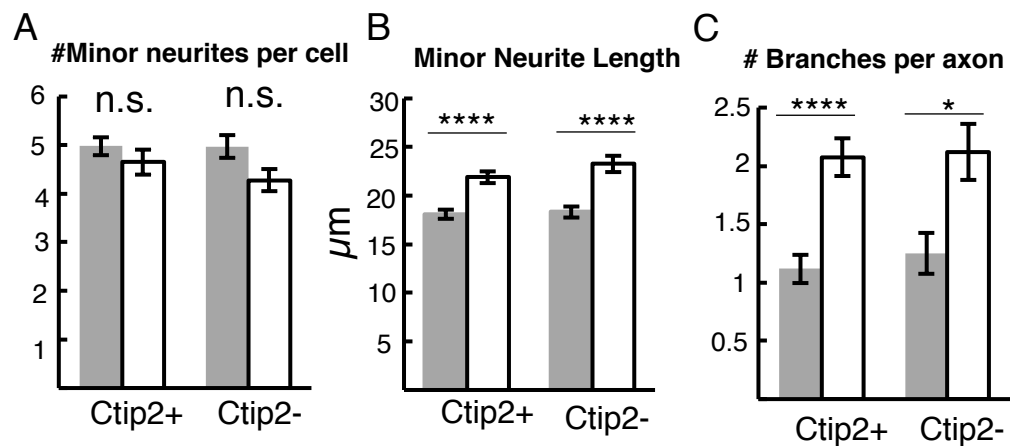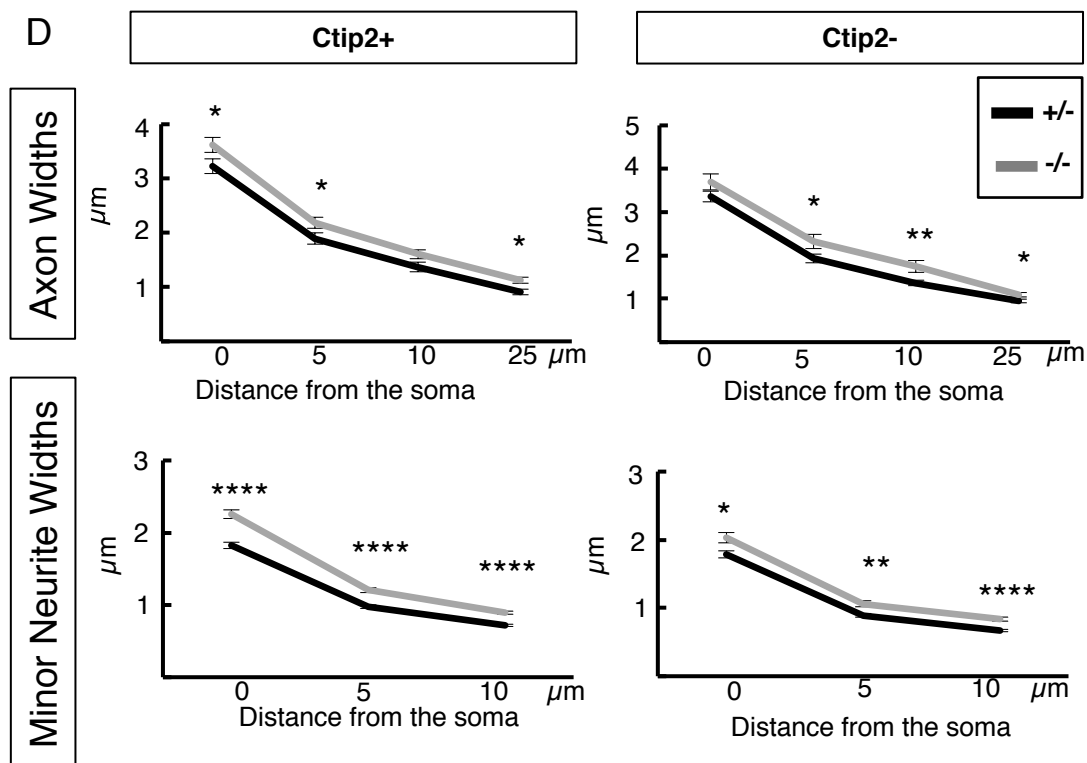

Supplement: Additional file 1: Figure S1. — Morphological abnormalities in Kif20b mutant cells are observed in both Ctip2+ and Ctip2- neurons. A. The average number of minor neurites is not different in Kif20b mutant cells (white bars) whether Ctip2- or Ctip2+. B. Minor neurites are longer on average in Kif20b mutant neurons (white bars) than in control neurons (gray bars), whether Ctip2+ or Ctip2-. C. Kif20b mutant axons (white bars) have more branches than control neurons (gray bars) whether Ctip2+ or Ctip2-. D. Axons and minor neurites of Kif20b mutant neurons are wider on average than controls, regardless of whether they are Ctip2+ or not. Measurements done using images of tubulin immunostaining at same exposure times. Ctip2+ neurons n = 43 +/- and 40 -/-. Ctip2- neurons n = 36 +/- and 25 -/-. Error bars are + s.e.m; *, p <0.05; **, p <0.01, ***, p <0.001, **** p <10-5, ***** p <10-7; n.s., not significant, t-test. (PDF 32 kb) [file 13064_2017_82_MOESM1_ESM.pdf]
